# Adolescents with cleft lip and palate (CLP): Stressful events and coping

**DOI:** 10.1192/j.eurpsy.2021.1936

**Published:** 2021-08-13

**Authors:** P. Pacheco, M. Pacheco, D. Molini-Avejonas, A. Mota-Loss

**Affiliations:** 1 Rehabilitation Science, USP, São Paulo, Brazil; 2 Morphology, UFES, Espirito Santo, Brazil; 3 Speech Language Pathology And Audiology, UFES, Espirito Santo, Brazil

**Keywords:** Cleft lip and palate, adolescence, coping, stress

## Abstract

**Introduction:**

Individuals with CLP may present communication disorders, velopharyngeal dysfunction, dento-occlusal changes and hearing losses. Adolescents with CLP usually show greater impairment of communication. Such characteristics combined with the malformed face can impact psychosocial functioning and increase the risk of psychological difficulties. Life-stressing experiences from CLP to life events typical of adolescence, may threaten the well-being of the adolescent, and are linked to mental health and behavioral problems.

**Objectives:**

To verify the coping of adolescents with CLP through a descriptive cross - sectional study.

**Methods:**

Fifteen adolescents with CLP participated in the study. To evaluate them it was used the Coping scale (Lees, 2007), for the verification of coping in the families proposed by Motivational Theory of Coping. The analysis of the coping of adolescents with CLP considered two adverse contexts, namely “birth with fissure” and “have your secret told by a friend ”.

**Results:**

In relation to the evaluation of the psychological needs of relationship, competence and autonomy faced the “birth with fissure” indicates that teens with fissures do not perceive this stressor as a threat to their basic psychological needs.

**Conclusions:**

The adolescents with CLP who participated in the study feel more interested (perception of the challenge) in dealing with the stressor relative to the fissure than in dealing with the betrayal of a friend, who reveals a secret of his to other people.

**Disclosure:**

No significant relationships.

